# Supplementary material for: Atomistic Study on the Sintering Process and the Strengthening Mechanism of Al-Graphene System
Source: Materials (Basel). 2022 Apr 4;15(7):2644. doi: 10.3390/ma15072644 (PMC9000460; doi:10.3390/ma15072644)
Supplement: Supplementary file 1 [file materials-15-02644-s001.zip › materials-1640480-supplementary.pdf]

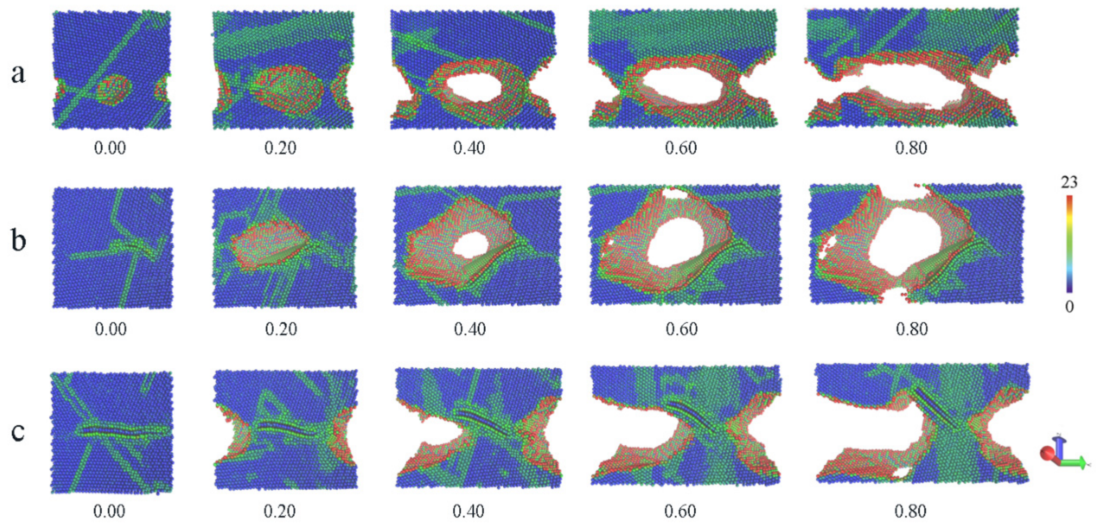

Figure S1. The evolutions of atomic structures under different strains when stretched in plane direction. (a) the pure Al structure, (b) the structure of composite with SLG, (c) the structure of composite with BLG.

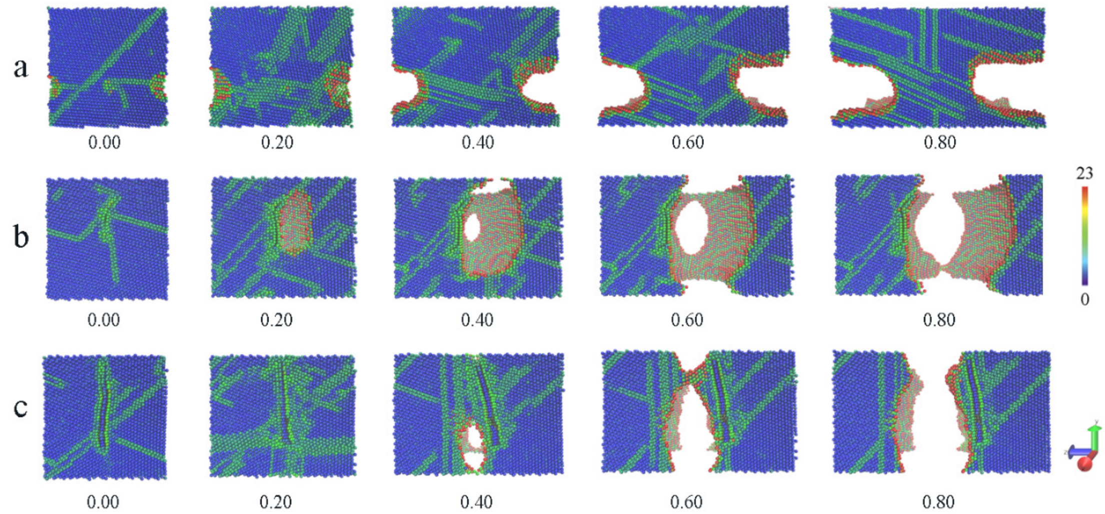

Figure S2. The evolutions of atomic structures under different strains when stretched in normal direction. (a) the pure Al structure, (b) the structure of composite with SLG, (c) the structure of composite with BLG.
